# A new advanced cellular model of functional cholinergic-like neurons developed by reprogramming the human SH-SY5Y neuroblastoma cell line

**DOI:** 10.1038/s41420-023-01790-7

**Published:** 2024-01-12

**Authors:** Alessia D’Aloia, Valentina Pastori, Stefania Blasa, Gloria Campioni, Francesco Peri, Elena Sacco, Michela Ceriani, Marzia Lecchi, Barbara Costa

**Affiliations:** 1 Department of Biotechnology and Biosciences, University of Milano-Bicocca, Piazza della Scienza 2, 20126 Milano, Italy; 2 Milan Center for Neuroscience (NeuroMI), University of Milano-Bicocca, Piazza dell’Ateneo Nuovo 1, 20126 Milano, Italy; 3 SYSBIO-ISBE-IT, Europe, 20126 Milano, Italy; 4 Inter-University Center for the Promotion of the 3Rs Principles in Teaching & Research, Pisa, Italy

**Keywords:** Differentiation, Cellular neuroscience

## Abstract

Modeling human neuronal properties in physiological and pathological conditions is essential to identify novel potential drugs and to explore pathological mechanisms of neurological diseases. For this purpose, we generated a three-dimensional (3D) neuronal culture, by employing the readily available human neuroblastoma SH-SY5Y cell line, and a new differentiation protocol. The entire differentiation process occurred in a matrix and lasted 47 days, with 7 days of pre-differentiation phase and 40 days of differentiation, and allowed the development of a 3D culture in conditions consistent with the physiological environment. Neurons in the culture were electrically active, were able to establish functional networks, and showed features of cholinergic neurons. Hence here we provide an easily accessible, reproducible, and suitable culture method that might empower studies on synaptic function, vesicle trafficking, and metabolism, which sustain neuronal activity and cerebral circuits. Moreover, this novel differentiation protocol could represent a promising cellular tool to study physiological cellular processes, such as migration, differentiation, maturation, and to develop novel therapeutic approaches.

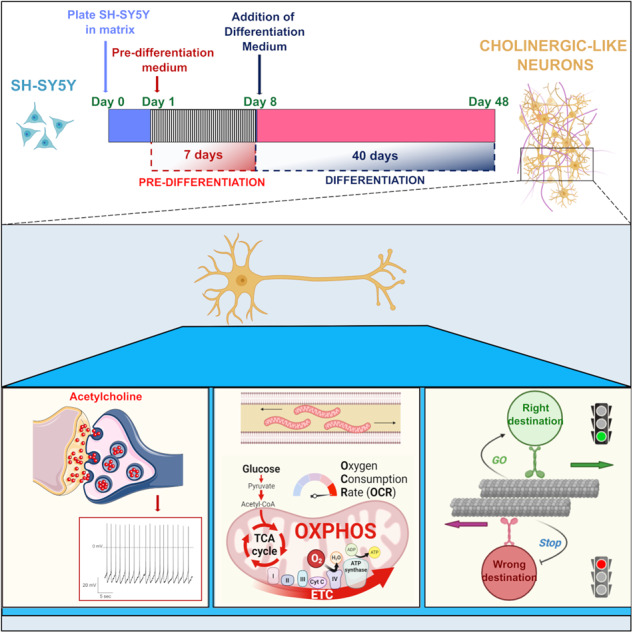

## Introduction

The cellular architecture of the human nervous system provides the foundations of our cognitive skills and disease susceptibility. In particular, the cerebral cortex is the most complex structure known in biology. Although base architecture seems to be preserved across mammals [1], different studies have suggested significant differences in the cellular composition of the human nervous system [2]. Specifically, marked variations were observed in the proportions of neuronal and non-neuronal cells and in the transcription of genes associated with neuronal structure and activity [3]. These species-specific discrepancies are reflected in neuronal function and raise doubts about using in vivo murine models to study neuronal disorders, microenvironment, and drug design. Nowadays, human neuron usage is restricted by ethical and technical reasons [4–7], thus different immortalized cell lines are used as alternatives. The main problem with the use of cell lines is that, currently, there are not any relevant in vitro models able to reflect the functional activity of mature neurons; for this reason, many areas in the field of neuroscience are hindered by this missing. Human induced pluripotent stem cells (iPSCs) are currently the most used approach to modeling human neuronal microenvironment in physiological and pathological conditions to discover and screen new potential drugs [8]. The possibility to fully reprogram cells from patients with neurological disorders to obtain neuronal cultures has allowed neuroscientists to recapitulate in the dish phenotypes associated with a particular disease [9–12]. However, although iPSCs are extensively used for the discovery and validation of new pharmacological treatments without ethical restrictions, human neuronal cultures achieved after differentiation in vitro have not been updated to reflect fundamental principles of brain physiology. Moreover, incomplete cellular reprogramming and the genetic and epigenetic changes that can occur with prolonged culturing of iPSCs further limit the usage of this in vitro model [13–15]. iPSC-derived neurons (iPSCNs) show heterogeneous electrophysiological properties (action potential firing frequency, action potential amplitude, resting membrane potential). Several reports have demonstrated that the resting membrane potential of human iPSCN hyperpolarizes over prolonged periods in culture and can reach relatively mature values after several months [16]. However, in standard conditions, some populations of iPSC neurons generate spontaneous activity at a very low frequency and only for a few days, limiting their use for short-term experiments [17].

Likewise iPSCs, neural stem cells (NSCs) are a promising approach to modeling neurological disorders, designing and screening new drugs, studying human embryonic neurogenesis, and providing the application in regenerative medicine. Induced pluripotent stem cells or embryonic stem cells are the most acceptable sources of NSCs [18]. However, to date, NSCs differentiation protocols drive the generation of a wide range of NSC phenotypes that exhibit different differentiation potentials and proliferative capacities [18]. Furthermore, many studies demonstrated that NSCs are more prone to differentiate into glial than neuronal phenotypes in systems reproducing pathological conditions [19]. This aspect, together with the lack of reproducibility and standardization of differentiation protocols, makes the use of NSC lines critical for disease modeling and even more so for cell therapy.

The most common immortalized human cell line used to obtain neuronal culture in vitro is SH-SY5Y. SH-SY5Y is a subclone of the SK-N-SH neuroblastoma that, depending on treatments/culture conditions, can be differentiated into various neuronal phenotypes. Currently, the most used agents to differentiate these cells include retinoic acid (RA) [20], administered in different concentrations and incubation times, and neurotrophic factors such as nerve growth factor (NGF), brain-derived neurotrophic factor (BDNF), neuregulins, GLP-1, and phorbol esters [21–23]. Furthermore, several methods to improve neuronal differentiation involve the usage of an extracellular matrix (ECM) gel to reproduce a three-dimensional (3D) environment [24–26]. The advantages of the adoption of differentiated SH-SY5Y cells as a neuronal model comprise large-scale amplification before differentiation, low cost, straightforward to culture compared to primary neurons, expression of human-specific proteins and their isoforms that would not be genetically present in murine primary cultures, and achievement of a homogenous neuronal cell population [21, 27, 28]. However, electrophysiological analyses are scarce and show that the functional properties of differentiated SH-SY5Y cells do not correlate with morphological differentiation and the expression of markers of neuronal maturation. In the majority of papers that functionally investigated differentiated SH-SY5Y cells, electrical activity was limited to induced action potentials, which were discharged at low frequencies [29–32], whereas a faint spontaneous spiking activity has been shown only by one research group [33, 34]. However, the characterization and quantification of this activity have not been performed.

Since the presence of neuronal electrical properties is the core of nervous system activity, here we report a new differentiation protocol to obtain a 3D culture of electrically functional cholinergic-like neurons, starting from a readily available human neuroblastoma cell line. Hence we provide an easily accessible, reproducible, and reasonable technique that empowers studies of neurophysiological activity, synaptic function, vesicle trafficking, metabolism, and events that play a critical role in neuroscience. The improvements made in this differentiation protocol could narrow the gap between in vitro neuronal models and in vivo neuronal physiological conditions. Furthermore, improving electrical and synaptic activity in vitro allows for modeling the physiological behavior of neuronal networks, from which to derive neurological disorders in a dish with more realistic conditions.

## Results

### 3D DMAP2 Mix condition induces functional differentiation in SH-SY5Y cells

**Fig. 1:**
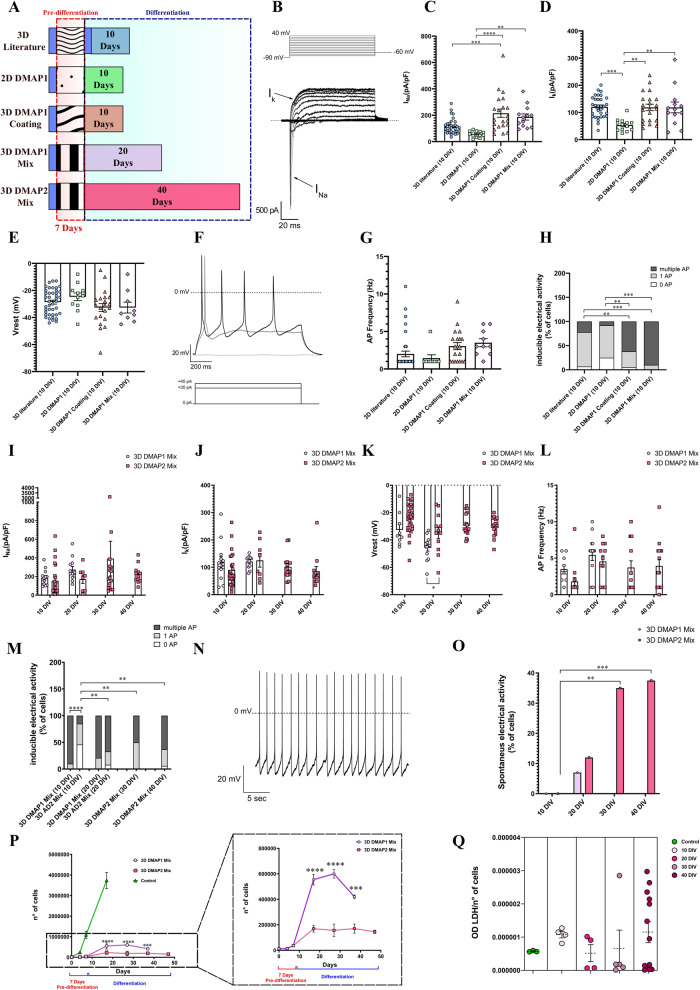
Functional properties and viability of SH-SY5Y cells cultured in different 2D and 3D conditions at different days of differentiation. **A** Schematic representation of diverse protocols used to promote differentiation in SH-SY5Y cells (described in the Materials and Methods section). The main electrophysiological parameters of a mature neuron were measured in cells at 10 DIV; they were: densities of sodium and potassium currents evoked by a standard voltage protocol (**B**, **C**, **D**), resting membrane potential (**E**), and induced action potential firing frequency (**F**, **G**, **H**). The same parameters were measured for the conditions 3D DMAP1 Mix and 3D DMAP2 Mix, which turned out to be promising for inducing SH-SY5Y functional differentiation. (**I**–**M**) Evaluation of the different electrophysiological parameters at 10, 20, 30, and 40 DIV. **N** Representative spontaneous activity recorded from a differentiated SH-SY5Y cell. The percentage of cells with spontaneous activity in the 3D DMAP2 Mix increased up to 37% at 40 DIV (**O**). **P** SH-SY5Y cells were plated on 24‐well plates in the Growth Factor Reduced (GFR) basement membrane matrix. After 24 h, cells were differentiated using the 3D DMAP1 Mix or 3D DMAP2 Mix protocols, or left undifferentiated (Control). The number of viable cells was determined at 4, 7, 17, 27, 37, and 47 days for 3D DMAP1 Mix and 3D DMAP2 mix cultures; and at 4, 7, and 17 days for control cultures. The numbers of total samples analyzed for each time point described above were: 3, 4, 13, 2, and 4, samples for the 3D DMAP1 Mix condition; 3, 4, 11, 3, 3, and 13 samples, for the 3D DMAP2 Mix condition samples; 4 samples for the control condition. **Q** SH-SY5Y cells were plated on 96-well plates and differentiated as described in the Materials and Methods section (3D DMAP2 Mix) or left undifferentiated (Control). Cell cytotoxicity was quantified after 10, 20, 30, and 40 days of differentiation or after 7 days in culture for the control sample. The numbers of samples analyzed for each time point in two independent experiments were: control (*n* = 3), 10 DIV (*n* = 4), 20 DIV (*n* = 4), 30 DIV (*n* = 5), and 40 DIV (*n* = 13). Further details are described in the Materials and Methods section. Statistical tests used to determine the significance of differences among the conditions were: One-way ANOVA followed by Tukey’s multiple comparisons test, for data in panels **C**, **D**, **H**, and **Q**; Multiple t-tests, in panels **K**, **M**, and **P** (3D DMAP1 vs 3D DMAP2); Chi-Square test, in panel **O**. Significance is indicated as *p < 0.5, **p < 0.01, ***p < 0.001, ****p < 0.0001.

An electrophysiological investigation was performed to evaluate the functional properties of SH-SY5Y cells in the different culture conditions we applied to promote neuronal differentiation (Fig. 1A and Materials and Methods section). Cells were analyzed by studying the main parameters related to the electrical activity of differentiated neurons, such as current densities through voltage-dependent Na^+^ and K^+^ channels, resting membrane potential, and action potential firing frequency, which are representative of the intrinsic neuronal membrane properties. Na^+^ and K^+^ currents were recorded using the protocol described in the Materials and Methods section and were identified as inward transients (I_Na_) and outward steady-state signals (I_K_) respectively (Fig. 1B). The maximum values of current intensity recorded by each cell were normalized on cell capacitance to obtain current densities. Action potential frequencies were calculated on the activity generated by the cell in response to depolarizing currents injected by the patch micropipette. On the contrary, the ability to generate spontaneous activity was recorded without any current injection; this parameter was also investigated because it reflects the establishment of synaptic connections in the neuronal networks.

Results showed that, at 10 DIV, 3D cultures had significantly higher Na^+^ and K^+^ current densities compared to 2D cultures. In particular, the 3D Coating cultures had higher Na^+^ current density compared to 3D cultures prepared as described in the literature (Fig. 1C, D). Moreover, cells in 3D cultures showed a trend to have more hyperpolarized resting membrane potentials (Fig. 1E) and higher action potential firing frequencies compared to 2D cultures (Fig. 1F, G). Furthermore, 3D Coating and 3D Mix cultures exhibited a significantly higher percentage of cells with multiple induced action potentials (Fig. 1H). Thus, 3D Coating and 3D Mix cultures were maintained until 15 DIV, when the 3D Mix cultures showed an improvement in the overall functional properties compared to 3D Coating cultures (Supplementary Fig. S1A-E). 2D cultures were analyzed starting from 5 DIV (as described in the literature) until 10 DIV. Data showed that over this culture period, the action potential firing frequency and the ability of cells to generate multiple action potentials showed a decreasing trend (Supplementary Fig. S1F-J), and for this reason, this condition was excluded. 3D cultures described in the literature were analyzed at 10 and 20 DIV and results showed no significant differences over this culture period, suggesting that a stationary state in the electrophysiological properties was reached by the cells. Moreover, their phenotype was less differentiated compared to that of 3D Coating and 3D Mix cultures; thus, this condition was not investigated further (Supplementary Fig. S1K-O).

Since these results indicated that 3D culture conditions were promising for inducing SH-SY5Y functional differentiation, further electrophysiological characterizations of these cultures at different times of differentiation were performed (results at 10, 20, 30, and 40 DIV are shown in Fig. 1I, M; at 25 DIV in Supplementary Fig. S1R-W). Cells in the DMAP1 Mix condition seemed to exhibit a differentiated phenotype; however, their viability decreased after 20 DIV. Thus, the DMAP2 Mix condition was developed to try to maintain cultures for longer periods. This condition allowed an improvement in cell functional properties up to 40 DIV compared to 10 DIV. In fact, in the DMAP2 Mix condition, spontaneous action potentials could be recorded starting from 20 DIV (Fig. 1N), and the percentage of cells with spontaneous activity increased from 12% at 20 DIV to 37% at 40 DIV (Fig. 1O). Furthermore, no detrimental effects on cell viability and cytotoxicity were found in this condition (Fig. 1P, Q, Supplementary Fig. S1V). Besides, in the DMAP2 Mix condition, cells stopped growing from 10 DIV (indicated in the graph as 17 days, which corresponds to 7 days of pre-differentiation followed by 10 days of differentiation) reaching a plateau that held up to 40 DIV (indicated in the graph as 47 days) (Fig. 1P). In particular, starting from 10 DIV (17 days), there were significantly more cells in the DMAP1 Mix group than in the DMAP2 Mix group; the difference was maintained until 20 DIV when the viability of the DMAP1 Mix dropped (Fig. 1P). Taken together these results suggest that the DMAP2 Mix could be a promising culture condition to induce functional neuronal differentiation in SH-SY5Y cells.

### SH-SY5Y cells in 3D DMAP2 Mix condition express neural markers and display complex neuritic arborization before functional maturation

**Fig. 2:**
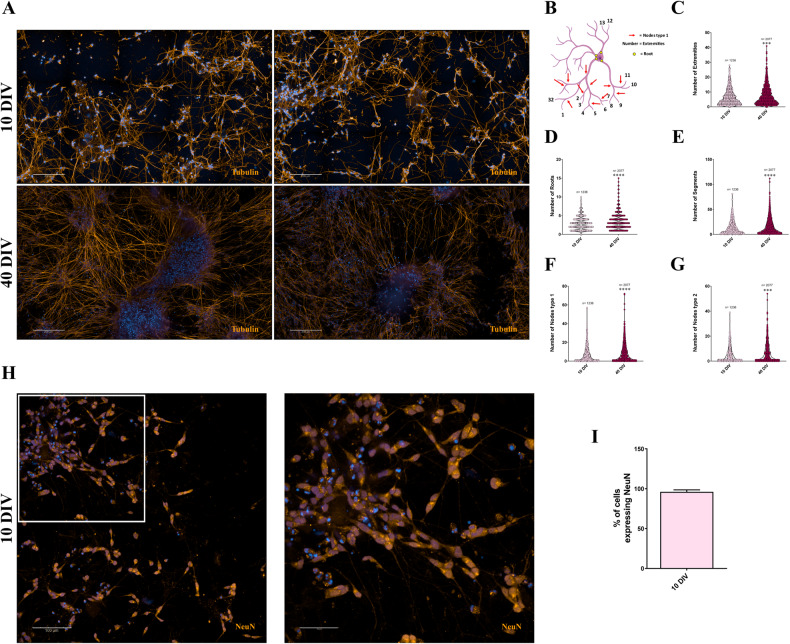
Morphological analysis revealed complex neuronal networks and high expression of NeuN in SH-SY5Y cells cultured in 3D DMAP2 Mix at 10 DIV and/or 40 DIV. **A** SH-SY5Y cells were plated and differentiated in 3D DMAP2 Mix condition as described in the Materials and Methods section. Cells were fixed, permeabilized, and immunostained with either the anti-β-tubulin (orange) antibody and Hoechst (blue) to detect neurite networks and nuclei, respectively. A total of 10 z-stacks images for each condition were taken. Maximum projection and large images (6 fields) are shown. Scale bar: 200 µm. **B** Schematic representation of some parameters taken into account during neuronal arborization analysis. In particular, the main parameters measured were: the number of extremities (**C**), the number of roots (**D**), the number of segments (**E**), the number of nodes type I (**F**), and the number of nodes type II (**G**). **H** The panel illustrates the NeuN (orange) and Hoechst (blue) merged staining at 10 DIV; the overlay is represented in pink. A total of 10 z-stacks images for each condition were taken. Maximum projection and large images (4 fields) are shown on the left. Scale bar: 100 µm. One representative field is shown on the right. Scale bar: 50 µm. **I** Quantification of NeuN positive cells (histogram); 95 positive neurons out of 99 neurons analyzed from 12 different fields. The totality of fluorescence images was captured using Operetta CLS™ equipped with a 63× immersion objective. Mann-Whitney test was used to determine the significance of differences among the conditions analyzed. Significance was set as *p < 0.5, **p < 0.01, ***p < 0.001, ****p < 0.0001.

To investigate the complexity of neuronal networks during differentiation with 3D DMAP2 Mix, cultures were immuno-labeled with an antibody against β-tubulin. In particular, the totality of cells expressed β-tubulin since 10 days of differentiation (10 DIV), mainly in the cell body and the leading neurite. With time in culture, notably after 40 days of differentiation (40 DIV), all cells displayed more complex neuritic arborizations (Fig. 2A). Figure 2B summarizes some of the parameters considered in our study to quantify this aspect. This analysis showed an improvement in the numbers of neurites (roots), branching nodes (type I nodes), branching (segments), terminal nodes (extremities), and trees (type II nodes) between 10 and 40 DIV (Fig. 2C–G). Appropriate growth and branching of neurites are essential for neurons and nervous system functions. Neurite development determines the number and the pattern of synapses received by each neuron [35]. Therefore, designing a differentiation protocol that promotes neurite arborization is essential for studies directly focusing on neuronal morphology and potentially critical for disease-modeling studies. Defects in neurites outgrowth are often associated with a severe neurodevelopmental disorder [35]. Digital phase contrast (DPC) images of live cells at different differentiation stages were analyzed to further demonstrate the improvement of neural network complexity during differentiation. In particular, an increase in process length (µm) per cell was tracked by monitoring each large image (25 fields) considered (6 large images) in the analysis during differentiation (Supplementary Fig. S2). Moreover, the increase in differentiation time matched the enhancement in process length (Supplementary Fig. S2). Time-lapse imaging of living cells at 10 and 40 DIV confirmed this data (Video 1 and 2) and highlighted changes in neurites dynamics, consistent with a physiological neuronal maturation. During 10 days of differentiation, neurites increased rapidly and were more dynamic; afterward, they grew more slowly, and around 40 DIV became structurally stable. Neurite formation has been described to occur in three stages: protrusion, arborization, and consolidation [36, 37]. The switching in neurite growth rate and arbor dynamics from stage 2 to stage 3 corresponds to improved synaptic establishment and strength in neurons. The data support the idea that weaker synaptic inputs, as suggested at 10 DIV by electrophysiological recordings, allow a greater degree of dynamic rearrangements and a faster growth rate in the neurite arbor; otherwise, strong synaptic inputs, as observed at 40 DIV, stabilize neurite arbor structures and decrease arbor dynamism [37]. Finally, to extend and more precisely characterize differentiating cells, cultures were immuno-labeled with an antibody against the neuronal-specific nuclear marker NeuN. Differentiated SH-SY5Y stained positively for NeuN (~96%, 95 out of 99 neurons analyzed from 12 different fields) already at 10 DIV (Fig. 2H, I). These results, together with β-tubulin-staining, confirm that the expression of neuronal markers arises before the acquisition of the complete functional maturation.

### SH-SY5Y cells in 3D DMAP2 Mix condition express synapse-specific proteins and develop mature synaptic ultrastructures

**Fig. 3:**
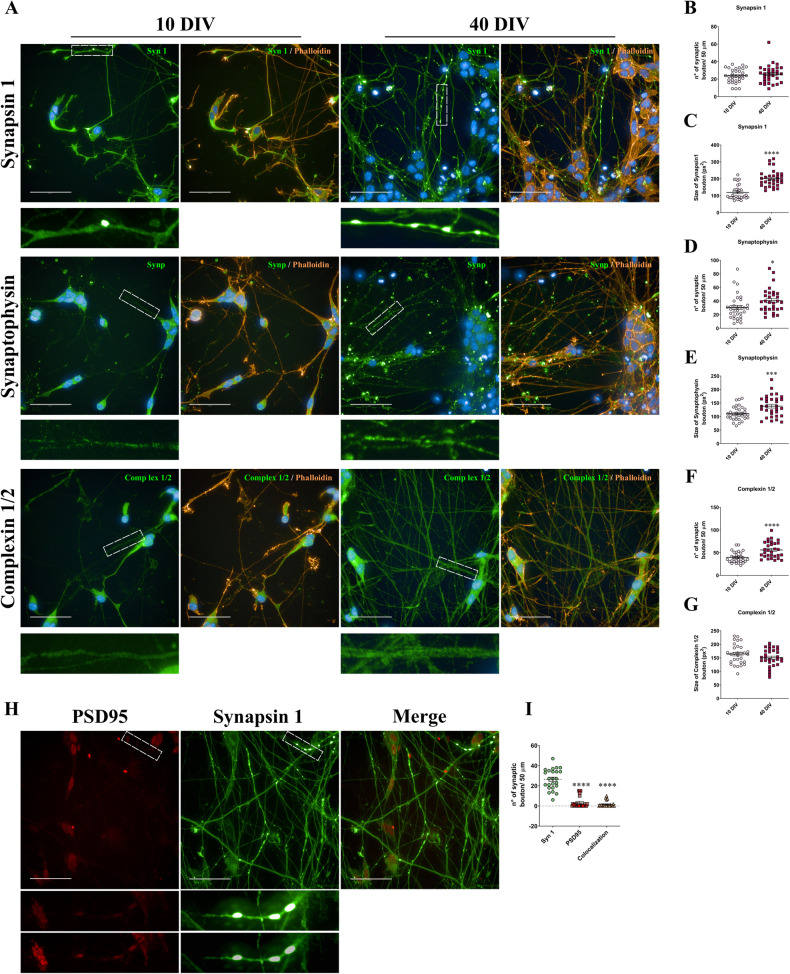
Expression of pre and postsynaptic markers. **A** SH-SY5Y cells were plated and differentiated as described in the Materials and Methods section (3D DMAP2 Mix). Cells were fixed, permeabilized, and immunostained with anti-synapsin 1 (green) or anti-synaptophysin (green), or complexin 1/2 (green) in combination with TRITC-phalloidin (orange) and Hoechst (blue) to detect presynaptic density, actin, and nuclei respectively. A total of 10 z-stacks images for each condition were taken. Maximum projections are shown. Magnifications of 50 µm of neurites are also shown in the images below. Scale bar: 50 µm. The number and the size of synaptic boutons per 50 µm of neurites positive for (**B**, **C**) synapsin 1, (**D**, **E**) synaptophysin, (**F**, **G**) complexin 1/2 were determined. For Synapsin 1, at 10 and 40 DIV, *n* = 32 derived from 8 fields; for synaptophysin at 10 DIV, *n* = 33 derived from 8 fields, and at 40 DIV, *n* = 31 derived from 8 fields; for complexin 1/2 at 10 and 40 DIV, *n* = 30 derived from 8 fields. **H** Evaluation of the number of synaptic densities. Cells were immunostained with either anti-synapsin 1 (green) and anti-PSD95 to detect synaptic density. The color of PSD95-staining was changed from orange to red. A total of 10 z-stacks images for each condition were taken. Maximum projections are shown. Magnifications of 50 µm of neurites are shown in the images below (the first magnification derived from the original picture without any adjustment, while in the second one, the contrast was adjusted). Scale bar: 50 µm. **I** Number of synaptic boutons per 50 µm of neurites, positive for synapsin 1, PSD95, and for synapsin 1 and PSD95 in colocalization (*n* = 24 derived from 6 fields). The totality of fluorescence images was captured using Operetta CLS™ equipped with a 63× immersion objective. Differences among groups were tested for significance by: Unpaired t-test, in panels **B**–**G**; One-way ANOVA followed by Tukey’s multiple comparisons test in panel **I**. Significance was set as *p < 0.5, **p < 0.01, ***p < 0.001, ****p < 0.0001.

The capability of a neuron to form synaptic connections is an essential requisite for functional maturation. Therefore, the expression of presynaptic markers and quantification of pre-synaptic puncta were investigated. Cultures at 10 and 40 DIV were immune-labeled with antibodies against synapsin 1, synaptophysin, or complexin 1/2. Until 10 DIV, the totality of cells expressed presynaptic markers mainly in the soma and the neurites (Fig. 3A). At a longer time, notably after 40 DIV, the entire culture displayed an increase in presynaptic protein localization at sites of neurite contacts (Fig. 3A). In particular, quantitative analysis revealed that synaptophysin and complexin 1/2 boutons number per 50 µm of neurites grew from 10 to 40 DIV while synapsin 1 puncta number did not change (Fig. 3B, D, F). Moreover, the size of synapsin 1 and synaptophysin boutons was enhanced from 10 to 40 DIV (Fig. 3C, E). To further study synaptic connections, we decided to quantify the number of pre-synaptic puncta per 0.043 mm^2^ area. Supplementary Fig. S3 shows that all presynaptic proteins considered in our study increased considerably from 10 to 40 DIV. These data are in agreement with the improvement in neural network complexity and the enhancement in process length we have observed during differentiation (Fig. 2A–G). Notably, synaptic maturation involves the increase of both synapse size and the amount of pre- and postsynaptic proteins [38]. Besides, despite the initial assembly of a synapse can be fairly rapid (just a few minutes), the establishment of a mature synapse is generally prolonged, as highlighted by the lag between the formation of mature ultrastructures [39] and the development of mature electrophysiological properties [38, 40–44]. Finally, we investigated the presence and the number of synaptic densities. In particular, synaptic density was assessed as colocalization between the pre- and post-synaptic markers synapsin 1 and PSD95 at 40 DIV. The totality of cells expressed PSD95 mainly in the cell body, whereas we observed weak synaptic staining within neurites or at sites of neurite contacts (Fig. 3H). Quantitative analysis revealed that the PSD95-positive puncta number was lower than the synapsin 1-positive puncta (Fig. 3I). Furthermore, we noticed that about 46% of PSD95-positive boutons are also synapsin 1-positive instead only about 6% of synapsin 1-positive boutons are also PSD95-positive (Fig. 3I), suggesting that PSD95 is weakly expressed in our neuronal cultures.

### SH-SY5Y cells generate vesicles with directional sorting trafficking in 3D DMAP2 Mix condition

**Fig. 4:**
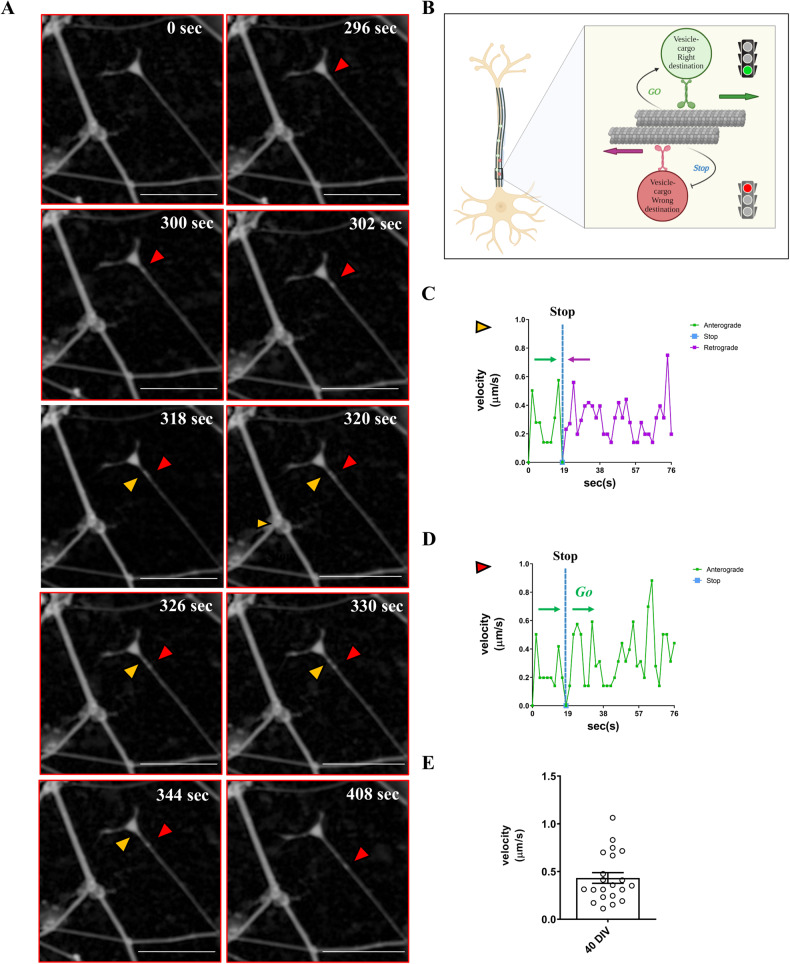
Vesicle trafficking. **A** SH-SY5Y cells were plated and differentiated as described in the Materials and Methods section (3D DMAP2 Mix). After 40 DIV, time-lapse imaging was performed using Operetta CLS™ at 63× magnification in Digital Phase Contrast (DPC) to detect neurites and vesicles. Magnifications of the time-lapse image at different time points are shown. The red arrow indicates the vesicle, which moved with an anterograde trajectory. In contrast, the yellow arrow indicates the vesicle moving with an anterograde trajectory, pausing, and reversing backwards to the soma. Scale bar: 30 µm. **B** Schematic representation of vesicle sorting check-point. Changing of velocity over time for vesicles marked with the yellow arrow (**C**) and red arrow (**D**). **E** The mean velocity was calculated for 21 vesicles (six neurons).

Vesicle trafficking was investigated using a single particle-tracking analysis. Digital phase contrast (DPC) live cell imaging of differentiating cells (40 DIV) clearly showed the presence of vesicles inside neurites. Time-lapse imaging of living cells revealed that these vesicles were carried through neurites with anterograde, retrograde, or stationary movement. Moreover, in the initial 20 µm segments of neurites, it was highlighted a peculiar behavior of certain vesicles: they moved with an anterograde trajectory, paused, and reversed backwards to the soma (~5%, 164 vesicles analyzed from 20 neurons) (Fig. 4A–D and Video 3). Karasmanis et al. demonstrated that due to the microtubule network of mixed polarity, membrane trafficking in dendrites appears without any capacity for directional sorting. Indeed, there is a clear check-point mechanism able to directional sorting vesicles. Entering into dendrites, axonally destined cargos pause and reverse backwards to the cell body, moving with a retrograde bias, while dendritically destined cargos are polarized in the anterograde direction [45]. A similar attitude was observed in the axonally sorting mechanism [46]. During entry into axonal, polarized sorting of somatodendritic and axonal vesicles occurs at the pre-axonal exclusion zone (PAEZ) and depends on the capability of vesicles to gain an appropriately directed microtubule motor protein [46].

To further study vesicle trafficking, functional vesicle motility along neurites was analyzed by taking pictures every 2 s for 10 min. Frame-by-frame analysis revealed that the mean velocity of vesicles was about 0.43 µm/s (Fig. 4E), in agreement with the literature [45]. Thus, during entry into neurites, vesicles undergo a sorting check-point mechanism. These data indicate that the proposed new differentiation protocol allows for a generation of cells with not only well-developed neuritic networks and synaptic structures but also with vesicles with directional sorting trafficking (Fig. 4B); this feature is important for the correct transmission of neural signals.

### Mitochondrial metabolism and morphology of SH-SY5Y cells are rearranged in 3D DMAP2 Mix condition

Since metabolic reprogramming is a key step of neuronal differentiation [47, 48], we analyzed cellular bioenergetics in SH-SY5Y cells subjected to our differentiation protocol, using the Seahorse eXtracellular Flux analyzer XFe96 (Agilent), which allows simultaneous real-time measurements on living cells seeded on 96 well-plates of oxygen consumption rate (OCR) and extracellular acidification rate (ECAR), parameters respectively related to mitochondrial and glycolytic capacity. In this work, we decided to focus on the analysis of mitochondrial bioenergetics, since the 3D structure of the cultures variably affects the buffer factor required for the calculation of the proton efflux rate (PER), a parameter directly proportional to lactate secreted in the culture medium, from the measured ECAR values also containing the contribution of CO_2_ produced by mitochondrial respiration.

**Fig. 5:**
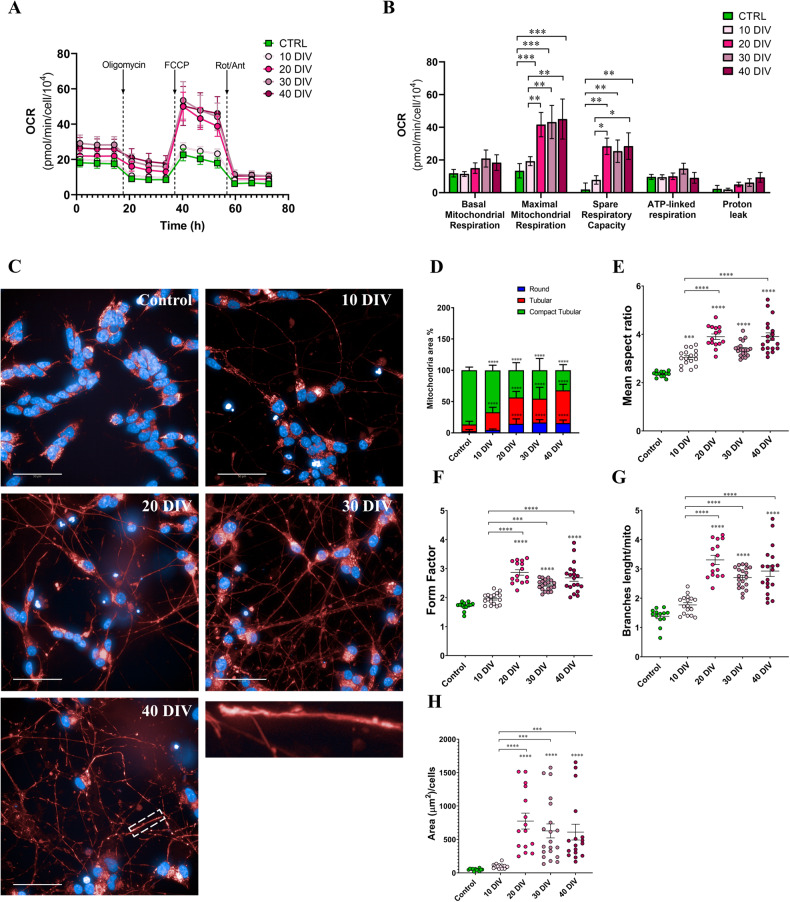
Metabolic characterization. **A** Representative Oxygen Consumption Rate (OCR) profile of SH-SY5Y cells cultured at different days of differentiation. The samples were subjected to XF Mito Stress Test with XFe96 Agilent Seahorse under sequential injections of 1.5 µM oligomycin A, 3 µM FCCP and 2 µM Rotenone + 2 µM Antimycin A. **B** Respiratory bioenergetics parameters measured from Seahorse results: Basal mitochondrial respiration, Maximal mitochondrial respiration, Spare respiratory capacity, ATP-linked respiration, and Proton leak. OCR values were normalized on cell number obtained by imaging analysis of nuclei stained with Hoechst 33342 immediately after the assay. *P* values were obtained by two-way ANOVA followed by Tukey’s multiple comparison test. The number of biological replicates analyzed, each one composed of at least 5 technical replicates, was: 3 for the control, 3 for 10 DIV, 3 for 20 DIV, 3 for 30 DIV, and 2 for 40 DIV. **C** Mitochondria morphologies. SH-SY5Y cells were plated and differentiated as described in the Materials and Methods section (3D DMAP2 Mix) or left undifferentiated. Live cells were stained with PhenoVue 641 Mitochondrial Stain (red) and Hoechst (blue) to detect mitochondria and nuclei respectively. Here, a total of 10 z-stacks images for each condition were taken and maximum projections are shown. Magnification of mitochondrion at 40 DIV is also shown in the last image. The totality of fluorescence images was captured using Operetta CLS™ equipped with a 63× immersion objective. Scale bar: 50 µm. **D** Harmony and PhenLogic machine-learning software were used to quantify the mitochondrial classes. Mitochondria were categorized as round area, long area, and compact tubular area. N values are reported in the main text or results. To corroborate data obtained by Harmony software additional analysis was performed by using Fiji software and the Mitochondria Analyzer plug-in. We analyzed (**E**) the mean aspect ratio, (**F**) form factor, (**G**) branches length/mito, and (**H**) total mitochondria area/cell. Control (*n* = 13 fields for panels **E, F, G,** while *n* = 12 panel **H**), 10 DIV (*n* = 17 fields), 20 DIV (*n* = 15 fields), 30 DIV (*n* = 20 fields), 40 DIV (*n* = 19 fields for panels **E**, **F**, **G**, while *n* = 17 panel **H**). Differences among groups were tested for significance by: Two-way ANOVA followed by Tukey’s multiple comparisons test in panel **D**; One-way ANOVA following by Tukey’s multiple comparisons test in panels **E**, **F**, and **G**; Kruskal-Wallis test followed by Dunn’s multiple comparisons test. Significance was set as *p < 0.5, **p < 0.01, ***p < 0.001, ****p < 0.0001.

To assess mitochondrial bioenergetics we used the mito stress test protocol consisting of the OCR measurement under basal conditions and after the treatment with different drugs, including the ATP synthase inhibitor oligomycin, the ETC accelerator ionophore FCCP, and an ETC inhibitors mixture (rotenone and antimycin A) (mito stress test profile, Fig. 5A), from which the mitochondrial parameters were calculated (Fig. 5B) as described in Materials and Methods Section.

Seahorse analysis highlighted a significant increase in maximal respiration and spare respiratory capacity in differentiated neurons starting from 20 DIV. No significant differences emerged in basal respiration, ATP-linked respiration, and proton leak. Notably from 20 to 40 DIV the maximal respiratory capacity is maintained at similar levels of oxygen consumption per cell suggesting that at 20 DIV cells have reached the mitochondrial rearrangement required for neuron maturation.

Mitochondria are highly dynamic organelles able to change their shape. In particular, mitochondria morphology is tightly related to their functionality. During neuronal differentiation mitochondria morphology changes from mixed globular and tubular structure to more elongated tubular structure, and this reflects the metabolic shift of cells from glycolysis to OXPHOS for an increase in bioenergetics [49]. Furthermore, the increase in mitochondria biogenesis is another important process that occurs during neural differentiation [47].

To further corroborate data obtained by seahorse analysis, mitochondria morphology was analyzed. We quantified mitochondrial phenotypes during differentiation using live-cell high-content-imaging analysis with PhenoVue 641 Mitochondrial Stain (Fig. 5C). In particular, we classified mitochondria as compact tubular, tubular, or round using Harmony and PhenLogic machine-learning software. Our analysis revealed that, during differentiation, there was a significant increase in the proportion of cells with a tubular mitochondria phenotype: 10.8 ± 1% control (25 fields analyzed, with about 30 cells for each field), 28.1 ± 1.9% 10 DIV (17 fields analyzed, with about 15 cells for each field), 42 ± 1.2% 20 DIV (67 fields analyzed, with about 19 cells for each field), 38.2 ± 3.5% 30 DIV (26 fields analyzed, with about 20 cells for each filed), and 52.5 ± 1.7% 40 DIV (30 fields analyzed, with about 15 cells for each filed) (Fig. 5C and D). Additional analysis using Fiji software and the Mitochondria Analyzer plug-in confirmed results obtained by Harmony software (Fig. 5E and F). Furthermore, during differentiation, we also observed a significant enhancement of branches length per mitochondrion (Fig. 5G) and an improvement in mitochondria biogenesis (Fig. 5H). Notably, all mitochondria parameters calculated (shape, network, and mitochondria biogenesis) were maintained at similar levels from 20 to 40 DIV as observed in seahorse analysis.

### SH-SY5Y cells differentiate towards a cholinergic-like phenotype in 3D DMAP2 Mix condition

**Fig. 6:**
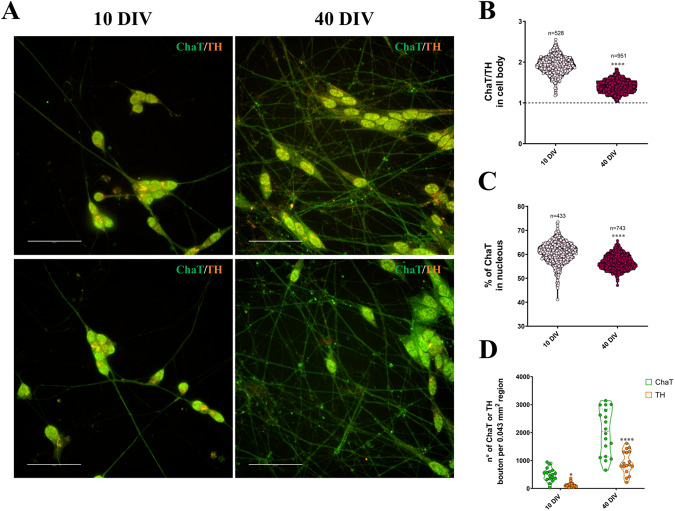
Characterization of neuronal phenotype. **A** SH-SY5Y cells were plated and differentiated as described in the Materials and Methods section (3D DMAP2 Mix). Cells were fixed, permeabilized, and immunostained with either the anti-ChaT (green) or the anti-TH (orange) antibodies. A total of 10 z-stacks images for each condition were taken. Maximum projections of overlay are shown. The totality of fluorescence images was captured using Operetta CLS™ equipped with a 63× immersion objective. Scale bar: 50 µm. Quantification of (**B**) the ChaT/TH ratio in the cell body at 10 DIV (*n* = 528 cells) and 40 DIV (*n* = 951 cells). **C** The percentage of ChaT in the nucleus at 10 DIV (*n* = 433 cells) and 40 DIV (*n* = 743 cells) was performed using Harmony software, while quantification of (**D**) the number of ChaT and TH vesicles in neurites per 0.043 mm^2^ region at 10 DIV (ChaT *n* = 17 fields, TH *n* = 18 fields) and 40 DIV (ChaT *n* = 18 fields, TH = 17 fields) was performed using Fiji software. Statistical tests used to determine the significance of differences among the conditions were: Unpaired t-test, in panels **B** and **C;** Multiple t-tests, in panel **D.** Significance, was set as *p < 0.5, **p < 0.01, ***p < 0.001, ****p < 0.0001.

Depending on culture conditions, SH-SY5Y cells can be differentiated into several adult neuronal phenotypes, including cholinergic, dopaminergic, or adrenergic [50]. Undifferentiated SH-SY5Y cells express low levels of both endogenous tyrosine hydroxylase (TH) and choline acetyltransferase (ChaT), enzymes involved respectively in dopamine (DA) and acetylcholine (ACh) synthesis [28]. To figure out which neuronal phenotypes our differentiation protocol can drive, cultures at 10 and 40 DIV were immune-labeled with antibodies against TH and ChaT. As shown in Fig. 6A, since 10 DIV, the totality of cells expressed both TH and ChaT, mainly in the cell body and the leading neurites. In particular, ChaT showed a nuclear localization both at 10 and 40 DIV. With time in culture, notably after 40 DIV, all cells displayed an increase of ChaT localization within neurites and at the sites of neurite contacts. Quantitative analysis revealed that the expression of ChaT in cell bodies was higher than TH-expression since 10 DIV, although this ratio decreased at 40 DIV (Fig. 6B). Furthermore, ChaT nuclear localization dropped from 10 to 40 DIV (Fig. 6C). ChaT expression reduction, both in the soma and nucleus at 40 DIV can be attributed to the sharp increase of ChaT localization within neurites at 40 DIV (Fig. 6D). Moreover, the size of ChaT-positive boutons was enhanced from 10 to 40 DIV, while TH puncta size decreased during differentiation (Supplementary Fig. S4). In general, we observed that in our cultures ChaT was more expressed than TH. Thus, we can conclude that neurons obtained during our differentiation protocol acquired a cholinergic-like phenotype.

## Discussion

The current lack of cellular models capable of recapitulating the characteristics of mature human neurons makes studies on neurodegenerative diseases and the design of new drugs for these pathologies very difficult/tricky. Specifically, the currently most promising cell models available (iPSCN, NSC) did not exhibit the electrical activity typical of a mature neuron, and the protocols developed to differentiate them are difficult to reproduce and standardize. Given the above, we developed a human 3D neuronal cell culture, starting from a readily available human neuroblastoma cell line, SH-SY5Y cells [51]. In this culture, neurons were able to establish functional networks, active synaptic structures, and vesicles with directional sorting trafficking, and showed properties of cholinergic neurons.

Several research groups have tried to differentiate SH-SY5Y by a combination of neurodifferentiative factors and scaffolds/gels to reproduce the extracellular matrix of the neural microenvironment [26, 52, 53]. Being inspired by the success in generating cells with morphological and biochemical characteristics of mature neurons [26], we designed a new culture protocol by combining three-dimensional culturing with the synergistic effects of multiple neurotrophic factors, such as Retinoic acid (RA), NGF, NRG1, VitD3, and BDNF. RA is a derivative of retinol (Vitamin A) which supports the differentiation of human neuroblastoma cells, iPSCs, and NSCs [54, 55]. The addition of retinoic acid to the cell culture medium is the most common procedure to induce neuronal differentiation. Many papers have demonstrated the efficacy of retinoic acid to derive neurons expressing phenotypic cholinergic or dopaminergic markers from SH-SY5Y cells [56–58]. The mechanism by which RA exerts its differentiative effect involves the switch from proliferation to differentiation by stimulating the expression of the nerve growth factor (NGF) receptor and by increasing cell sensitivity to the same factor [59]. NGF is a neurotrophin essential for neuronal differentiation and neurites outgrowth by activating the tyrosine kinase TrkA and the p75 receptors [60]. NGF-induced differentiation is caused by an increased mitochondrial remodeling, which regulates mitochondria biogenesis and metabolism and sustains mitochondrial mass, potential, and bioenergetics [61]. In fact, extensive metabolic reprogramming occurs during cell differentiation [47–49], and derivative metabolites play a key role in modulating the epigenetic status of cells, and thus altering gene expression patterns [62, 63]. In our work, seahorse experiments demonstrated that, during differentiation, SH-SY5Y cells acquire a greater mitochondrial respiratory capacity, which is reflected in a significant increase in FCCP-uncoupled maximal respiration, in mitochondrial mass and branching starting from 20 DIV. This functional metabolic rearrangement is in agreement with the enhancement of PGC1a-promoted mitochondrial biogenesis occurring during neural differentiation, which is extensively described in the literature [47]. It is noteworthy that right at 20 DIV, where the maximum mitochondrial respiration capacity and the maximum extension of the mitochondrial network have been reached, the cells behave as functionally mature neurons, capable of synthesizing acetylcholine.

NRG1 and Vitamin D are involved in neuronal differentiation, neurite outgrowth, neurotransmission, and in the synthesis of growth factors (i.e., NGF) and several neurotransmitters such as acetylcholine (ACh), dopamine (DA), and gamma-aminobutyric acid (GABA) [64–66]. In our cultures, the synergy of these compounds, together with the use of Growth Factor Reduced (GFR) basement membrane extract matrix and Optimem medium, enhances neuronal maturation, supporting differentiation instead of proliferation. In fact, GFR matrix provides components crucial for neuronal differentiation and high levels of brain extracellular matrix proteins, such as laminin. In our protocol, we set up a thin-layer (100-300 µm) 3D culture model. This thickness allows to perform analysis as in vitro cultures (e.g., real-time imaging, immunofluorescence, and biochemical assays), but mimicking the cytoarchitecture of brain tissue in its physiological environment. Furthermore, compared to 2D cell cultures, 3D cultures show longer viability, various differentiation patterns, longer neurite extension, and formation of higher-density networks [67–70]. Our data show that both 2D (2D DMAP1) and 3D Coating (3D DMAP1 coating) cultures were not able to reach complete neural maturation. In the first case, cells could establish 3D interactions neither with other cells nor with the extracellular matrix, whereas in the second case, although cells could assess interactions with the extracellular basement, they could not establish a 3D network. The use of Optimem medium to support neuronal differentiation and electrical and synaptic activity is one of the major novelties of our differentiation protocol. Hu et al. demonstrated that, compared to the conventional RPMI-1640 medium, the use of the Optimem improved minimum essential medium to differentiate PC12 cells, increased neurite length, adhesion rate, differentiation, expression of synapsin 1, and induced action potentials [71]. Here, we demonstrated that our method sustained the development of functional and active neuronal networks. By the approaches described in the literature, cells achieved a stationary state in the electrophysiological properties starting from 10 DIV; furthermore, the phenotype of cells treated with the literature approach (3D literature) was less differentiated concerning morphology, network extension, complexity, and especially for the absence of spontaneous activity. This could be caused not only by the lack of supplements in these cultures but also by the breaking of interactions established in the early stage of the differentiation process (pre-differentiation state) since the Agholme protocol required cells harvested by trypsinization to switch from the pre-differentiation (2D culture) to the differentiation phase (3D culture) [26]. Moreover, since the Optimem medium contains more hypoxanthine and thymine than other media, we supposed that these nutrients could sustain cell functional maturation. Purine and pyrimidine nucleotides are essential precursors for nucleic acid synthesis, but their functions are not limited to this [72], because purines act as metabolic signals, provide energy [73, 74], and participate in the synaptic process particularly associated with ACh, GABA, and glutamate neurotransmission [75]; whereas pyrimidines are involved in polysaccharide and phospholipid biosynthesis, detoxification processes [72, 76], and neurotransmission [77]. BDNF could support both cell proliferation [78] and differentiation [21, 26, 79]. Since the Agholme protocol also used BDNF instead of RA to induce cell differentiation, in our study the same protocol was employed to verify the eventual effects of this factor on SH-SY5Y cells. Although, in the early stage of differentiation, BDNF in 3D DMAP1 Mix seemed to support functional neuronal maturation, after 20 DIV cell viability decreased.

The synergistic effects of combining multiple factors allow us to obtain cells that show properties of cholinergic neurons. Choline acetyl-transferase (ChAT) and acetylcholine esterase (AChE), respectively involved in the synthesis and degradation of ACh, are markers of cholinergic neurons [80]. In neuronal cells, nuclear ChaT activates the transcription of selected genes, including the high-affinity choline transporter (CHT1) [81], which is responsible for the reuptake of choline in presynaptic terminals. Considering our model, at the early stage of the differentiating protocol (10 DIV), cells express ChaT mainly in the cell body and the leading neurites. Since at the beginning of the differentiation process cells need to synthesize CHT1, the amount of ChaT in the nucleus is higher than at the end of differentiation, when the synaptic activity is predominant and ChaT localizes mainly in the presynaptic terminal. In our cells, the nuclear localization of ChaT is reduced but maintained also at 40 DIV, and this reduction corresponds to an increase within neurites and at the sites of neurite contacts, with an enhancement of the size of ChaT-positive boutons. Moreover, we observed that our cultures expressed TH in addition to ChaT. Besides, Jeong et al. [82] have shown that about 70% of cholinergic neurons in the arcuate nucleus (ARC) of the mouse brain express both TH mRNA and the GABA synthesizing enzyme, glutamate decarboxylase, and vesicular GABA transporter transcripts. The key factor in human cholinergic mature neurons is synaptic density marker expression. In our cultures, at the beginning of the differentiation protocol, synapsin 1, synaptophysin, and complexin 1/2 were mainly expressed in the soma and the neurites while, when maturation was completed, they were principally localized at presynaptic terminus. This is fully in agreement with the increase in neural network complexity. On the other hand, to analyze the colocalization between the pre- and post-synaptic markers (synaptic densities) we used synapsin 1 and PSD95, but while synapsin 1 localized in neurite contacts, PSD95 presented weak staining within neurites. This data is not surprising since cholinergic neurons predominantly express PSD93, which is the paralog of PSD95 [83].

Electrophysiological properties of cholinergic neurons are quite heterogeneous depending on the brain region and the species they are isolated from [80]. For instance, adult mouse basal forebrain cholinergic neurons, serving as a primary source of cholinergic inputs to the cerebral cortex, have been differentiated into two identifiable subtypes, according to their excitability (“early” and “late” firing) [84]. Moreover, cholinergic neurons in the brainstem pedunculopontine nucleus have been divided into four groups for the presence or absence of the transient outward potassium A-current, low threshold spikes, and spike latency [85], and striatal cholinergic interneurons have shown diversity in their spontaneous firing frequency, which was related to the single (ACh) or dual (ACh and GABA) neurotransmitter release [86].

A detailed analysis of some electrophysiological parameters, extrapolated from our recordings from neurons in 3D DMAP2 Mix cultures at 30 and 40 DIV, showed that our data were consistent with the results obtained by Unal et al. [84], for adult mouse basal forebrain cholinergic neurons [84]. For instance, for the amplitude of the first action potential, the delay of the first action potential, and the amplitude of the after hyperpolarization, the values we calculated were: 69 ± 3 mV, 129 ± 32 ms, 12 ± 1 mV (*n* = 13–14 cells) *versus* 71 ± 9 mV, 107 ± 53 ms and 18 ± 10 mV, from Unal et al. [84]. This comparison supports that the electrophysiological parameters of neurons in our culture model were in line with the one described in the literature for certain subtypes of cholinergic neurons.

Cholinergic dysfunction has very severe and large effects on cognition and behavior [87]. Different patterns of alteration and/or degeneration of cholinergic neurons have been highlighted in various pathologies such as Alzheimer’s disease, Dementia with Lewy Bodies (DLB), Parkinson’s disease (PD), and atypical parkinsonian diseases (APD) and Huntington disease; further, a loss of cholinergic neurons has been found in dementias induced by exogenous causes (i.e Chronic alcohol abuse) [88, 89].

Since many cognitive dysfunctions have as common trait alterations in the cholinergic system, our novel differentiation protocol could represent an innovative procedure to induce functional maturation of neuronal cultures for the study of pathological models of neurodegeneration, such as Alzheimer’s disease [90]. Of course, to better mimic the complex neurophysiological environment, it will be of fundamental importance to set up a co-culture with the glial components. Glial cells are key modulators of neuronal function either in physiological conditions or in neurodegenerative diseases [91, 92]. The in vitro models available today are too sophisticated or lack physiological complexity, are expensive, or are based on murine cells and therefore do not have the specific characteristics of human neurons; moreover, in vivo models require high financial efforts, are time-consuming, and require the sacrifice of many animals. In this study, we developed and characterized a human 3D culture model of cholinergic-like neurons that can be used to study different aspects of cellular pathologies related to the functional impairment or degeneration of these neurons. This model is inexpensive, easily reproducible, and is based on human cells. Moreover, it is an ethical alternative to the use of animals by fully respecting the 3 R principles: Reduction, Refinement, and Replacement in product testing and scientific research [93]. Further physiological studies would provide a reliable investigation of the molecular mechanisms of this method; however, for therapeutic perspectives, this approach could represent a suitable model to study neuronal development and to investigate molecular mechanisms responsible for neurodegeneration.

## Materials and Methods

### Cell culture and differentiation

SH-SY5Y cells [94] were recently authenticated through genetic characteristic determination by PCR-single-locus-technology (Eurofins Genomics, Ebersberg, Germany). SH-SY5Y were cultured in RPMI-1640 supplemented with 10% fetal bovine serum (FBS, Gibco-ThermoFisher, Waltham, MA, USA), 2 mM glutamine (Euroclone, Pero, Italy), and 0.25% micozap prophylactic (Lonza, Walkersville, MD, USA) at 37 °C in a humidified atmosphere of 5% CO_2_. Cells were differentiated using the Agholme protocol [26] (3D Literature) or four new differentiation protocols. The first one was called 2D DMAP1 (D’Aloia Modified Agholme Protocol). In 2D DMAP1 cells were seeded at a density of 1×10^4^ cells/mL in a 35 mm dish (Corning) in RPMI-1640 with 10% FBS. The next day, the medium was changed to induce pre-differentiation. The pre-differentiation medium contains Optimem (Gibco-ThermoFisher, Waltham, MA, USA) supplemented with 10 µM retinoic acid (RA) (Merck Life Science, Darmstadt, Germany), 2 mM glutamine (Euroclone, Pero, Italy) and 0.25% micozap prophylactic; the duration of pre-differentiation phase was 7 days. Subsequently, the medium was replaced to induce differentiation. Differentiation medium contains Optimem with 50 ng/mL BDNF (ImmunoTools, Friesoythe, Germany), 10 ng/mL NGF (ImmunoTools, Friesoythe, Germany), 10 ng/mL neuregulin β1 (NRG) (ImmunoTools, Friesoythe, Germany), 24 nM vitamin D_3_ (VitD_3_) (Merck Life Science, Darmstadt, Germany), 2 mM glutamine and 0.25% micozap prophylactic. The duration of the differentiation phase was 10 days. The second one was called 3D DMAP1 Mix. In 3D DMAP1 Mix cells were harvested by Optimem with 2 mM glutamine, and the cell suspension was mixed with a pre-cooled Growth Factor Reduced (GFR) basement membrane matrix (ratio used for experiments 1:20) (Matrigel, Corning, New York, USA). Cells were seeded at a density of 1×10^4^ cells/mL in a 35 mm dish. The matrix was left to set at 37 °C until the day after. The next day, the medium was changed to induce pre-differentiation, as described above. In particular, the composition of the pre-differentiation medium and pre-differentiation duration were the same for all our design protocols. After 7 days, the medium was replaced to induce differentiation. The composition of the differentiation medium was the same described above for the 2D DMAP1 protocol. The duration of the differentiation phase was 20 days. The third one was called 3D DMAP1 Coating. This protocol is similar to 3D DMAP1 Mix; the only difference is that, in this case, the cell suspension was not mixed with the GFR matrix; but the dish was pre-coated with pre-chilled basement membrane matrix (1:20) and left to set at 37 °C until the day after. The next day, cells were seeded at a density of 1×10^4^ cells/mL in Optimem with 2 mM glutamine. After 24 hours, the medium was changed to induce pre-differentiation and differentiation as described for the 3D DMAP1 Mix. The duration of the differentiation phase was 10 days. The fourth one was called 3D DMAP2 Mix. The procedure for 3D DMAP2 Mix was the same as 3D DMAP1 Mix the only difference was the composition of the differentiation medium. In this case, after 7 days of pre-differentiation, the medium was not replaced, but NGF (10 ng/mL), NRG (10 ng/mL), and VitD3 (24 nM) were directly added to the pre-differentiation medium (containing 10 µM RA). The duration of the differentiation phase was 40 days. In all the protocols described above, the culture medium was changed every 3–4 days by removing 30% of the total volume and adding fresh medium corresponding to 40% of the total volume.

### Electrophysiological analysis

SHSY-5Y electrical activity was elucidated by using the Patch-Clamp technique in the whole-cell configuration through the pClamp8.2 software (pClamp, RRID:SCR_011323) and the MultiClamp 700 A amplifier (Axon Instruments; Molecular Devices, LLC, San Jose, CA, USA). The voltage-clamp mode was used to investigate currents through voltage-dependent sodium and potassium channels, whereas the current-clamp mode was used to measure resting membrane potential and to record induced and spontaneous electrical activity. Cells that did not generate spontaneous firing were depolarized with 1 s-current pulses, under conditioning hyperpolarization at -70 mV, to verify their capability to exhibit multiple and repetitive action potentials. The standard voltage protocol used to elicit sodium and potassium currents started from a holding potential of -60 mV, preconditioned the cells at -90 mV for 500 ms, and clamped the membrane at depolarizing steps from -80 to +40 mV, with 10 mV-increments. The standard extracellular solution, which culture medium has been replaced with, contained (mM): NaCl 135, KCl 2, CaCl_2_ 2, MgCl_2_ 2, Hepes 10, and glucose 5, pH 7.3. Recording pipettes of borosilicate glass had a resistance of 4 MΩ and were filled with a standard pipette solution, containing (mM): potassium aspartate 130, NaCl 10, MgCl_2_ 2, CaCl_2_ 1.3, EGTA 10, and Hepes 10, pH 7.3. In the voltage-clamp mode, resistance compensation was applied to obtain an error <10 mV. Experiments were performed at room temperature.

### Cell viability and cytotoxicity

#### Trypan blue exclusion assay

SH-SY5Y cells were seeded in 24-well plates (Euroclone, Pero, Italy) at a density of 1.2 ×10^4^ cells/well and were differentiated as described above (3D DMAP1 Mix, 3D DMAP2 Mix) or left undifferentiated (Control). At each time point (4, 7, 17, 27, 37, 47 days), cells were detached and counted as previously described [95]. In particular, in the trypan blue exclusion assay, we also considered pre-differentiation days (time points 4 and 7 days); therefore, differentiation days were expressed as progressive numbers (17, 27, 37, and 47 days).

#### LDH assay

Cells were seeded in 96-well plates (Euroclone, Pero, Italy) at a density of 1 × 10^3^ cells/well and were differentiated as described above (3D DMAP2 Mix) or left undifferentiated (Control). At each time point (10, 20, 30, and 40 days of differentiation), supernatants were collected (The control supernatant was collected 7 days after plating). According to the manufacturer’s instructions, the lactate dehydrogenase (LDH) levels released into the culture medium were quantified using the CytoTox 96® Non-Radioactive Cytotoxicity Assay kit (Promega). The data were expressed as the ratio between the optical density (OD) of LDH and the number of cells of each corresponding well.

#### MTT assay

Cells were plated and differentiated as described above (Cytotoxicity assay). At each time point (7 days of predifferentiation, and 10, 20, 30, and 40 days of differentiation) 10 µL of 3-(4,5-dimethylthiazol-2-yl)-2,5-diphenyltetrazolium bromide (MTT) stock solution (0.5 mg/mL; Sigma-Aldrich, St. Louis, MO, USA) was directly added to each well. Cells were incubated at 37 °C for 4 h after formazan crystals were dissolved as previously described [96].

### Antibodies and reagents

Mouse monoclonal anti-β-tubulin (used 1:150) was obtained from Sigma-Aldrich. Monoclonal anti-NeuN/Fox3 (used 1:150) produced in mouse primary antibody was purchased from Immunological Sciences. Rabbit monoclonal anti-Synapsin 1 (used 1:200), rabbit monoclonal anti-Synaptophysin (used 1:100), and rabbit monoclonal anti-Complexin 1/2 (used 1:800) were purchased from Cell Signaling (Synaptic Neuron Marker Antibody Sampler kit). Mouse monoclonal anti-PSD95 (used 1:100) was obtained from Cell Signaling. TRITC-labeled phalloidin (used 1:1000) was from Sigma Aldrich. Alexa Fluor 488 mouse anti-Choline Acetyltransferase (ChaT) (used 1:100) was obtained from Abcam. Mouse monoclonal anti-Tyrosine Hydroxylase (TH) (used 1:300) was purchased from Cell Signaling. Alexa Fluor 488 goat anti-rabbit (used 1:200), and Cy3 goat anti-mouse (used 1:200) were purchased from Life Technologies. PhenoVue 641 Mitochondrial Stain (used 100 nM) was obtained from PerkinElmer. Hoechst 33342 (working concentration 1 µg/mL) was purchased from ThermoFisher.

### Immunofluorescence and Operetta CLS™

SH-SY5Y cells were plated in 96-well plates (uClear, lid black, Greiner Bio-One, Kremsmünster, Austria) at a density of 1×10^3^ cells/well and were differentiated as described above (3D DMAP2 Mix). After 10 or 40 days of differentiation (DIV), cells were fixed with 3.7% paraformaldehyde (Sigma-Aldrich, St. Louis, MO, USA) in phosphate-buffered saline (PBS) (Euroclone, Pero, Italy) for 10 min, permeabilized for 4 min with 0.1% Triton X-100 in PBS, blocked with 1% bovine serum albumin (BSA) (Merck Life Science, Darmstadt, Germany) in PBS for 1 h, and stained with primary antibodies for 1 h at 37 °C, followed by secondary antibodies for 45 min at 37 °C and nucleus-stained with Hoechst 33342 for 10 min. Fluorescence images were captured with Operetta CLS™ (PerkinElmer, Inc, Waltham, MA, USA) equipped with a 63× immersion objective. Since this protocol allows to obtain 3D cultures, different z-stacks were captured at Operetta CLS™ and maximum projection was shown.

### Live-cell imaging and time-lapse

SH-SY5Y cells were seeded in 96-well plates (uClear, lid black, Greiner Bio-One, Kremsmünster, Austria) at a density of 1×10^3^ cells/well and were differentiated as described above (3D DMAP2 Mix or 3D DMAP1 Mix) or left undifferentiated (Control). *Neuronal network evaluation*. After 0, 7 days of pre-differentiation, and 10, 20, 30, and 40 DIV cell images were acquired using Operetta CLS™ equipped with a 20× immersion objective in Digital Phase Contrast (DPC) at 37 °C and 5% CO_2_. *Time-lapse neuronal network formation*. After 10 or 40 DIV time-lapse imaging was performed using Operetta CLS™ at 63× magnification as previously described [96, 97]. Images of each chosen field were captured every 5 min, in DPC, for 18 h at 37 °C and 5% CO_2_. *Vesicle trafficking*. After 40 DIV time-lapse imaging was performed using Operetta CLS™ at 63× magnification as previously described [96, 97]. Images of each chosen field were captured every 2 s, in DPC, for 10 min at 37 °C and 5% CO_2_. *Mitochondria transport and imaging*. After 10, 20, 30, and 40 DIV cells were stained (Control cells were stained one day after plating) with 100 nM PhenoVue 641 Mitochondrial Stain and Hoechst 33342 for 20 min. Before image acquisition, the staining medium was replaced with a fresh one without phenol red. Cell images were acquired using Operetta CLS™ equipped with a 63× immersion objective in fluorescence to detect mitochondria (PhenoVue 641 Mitochondrial Stain) and nuclei (Hoechst 33342).

### Seahorse

All the reagents and consumables were purchased from Agilent Technologies (Santa Clara, CA, USA) unless otherwise indicated. SH-SY5Y cells were seeded in Seahorse XF96 Cell Culture Microplates at a density 5 × 10^2^ cells/well and were differentiated as described above (3D DMAP2 Mix) or at a density of 5 × 10^4^ cells/well left undifferentiated (Control), in a volume of 200 μL/well. The day of the assay, the cells were washed twice 180 μL/well with Seahorse XF DMEM Medium, pH 7.4, supplemented with 10 mM D-Glucose, 2 mM L-Glutamine, and 1 mM Na-Pyruvate and finally, the cells were allowed to equilibrate in 180 μl/well of complete Seahorse XF medium for 1 h at 37 °C in a no-CO_2_ incubator. The assays were performed by means of Agilent Seahorse XFe96 Analyzer using the Mito Stress Test protocol.

The day before the assay, the XF Sensor Cartridge was hydrated with 200 µL/well of milliQ water and incubated overnight at 37 °C in a no-CO_2_ humidified incubator. The day after, the Seahorse XF Calibrant solution was used to replace milliQ water in the XF Sensor Cartridge, and the lyophilized drugs Oligomycin (150 µM), FCCP (100 µM) and Rotenone/Antimycin A (50 µM) were rehydrated in complete Seahorse XF DMEM Medium. The drugs were loaded in the corresponding ports of the sensor cartridge after being diluted 1:10 in complete Seahorse XF DMEM Medium, as indicated in Mito Stress Test Kit user guide, in order to reach the final concentrations of 1.5 µM Oligomycin, 3 µM FCCP, and 2 µM Rotenone/Antimycin A once injected into the Seahorse microplate. At the end of the Seahorse assay, Hoechst 33342 (H3570, Gibco-ThermoFisher, Waltham, MA, USA) was added to each well at a final concentration of 1 µg/mL, and after 15 min incubation at 37 °C, the images of nuclei were acquired by the Operetta CLS™ at 20× magnification. Seahorse parameters were normalized on the number of cells/well calculated using Harmony software, as reported previously [98, 99].

The analysis of respiratory parameters was performed using Wave 2.6.1 software and calculated using the following formulas:

- Basal respiration = OCR_Basal_ – OCR_Rot/AA_
- Maximal respiration = OCR_FCCP_ – OCR_Rot/AA_
- Spare respiratory capacity = OCR_FCCP_ – OCR_Basal_
- ATP-linked respiration = OCR_Basal_ – OCR_Oligomycin_
- Proton leak = OCR_Oligomycin_ – OCR_Rot/AA_

### Analysis of network complexity

The network complexity analysis was performed with Harmony software (PerkinElmer, Inc, Waltham, MA, USA). Cell segmentation was performed based on Hoechst and Tubulin staining to detect nuclei and neurites. Neurites were detected by using the Find Neurites building block. Neurite properties were calculated and parameters taken into account in our study were: the number of extremities, number of roots, number of segments, number of nodes type I, and number of nodes type II (corresponding to “number of segments” divided by “number of roots”) (Fig. 2B). The process length per cell (µm/cell) was calculated following the procedure previously described by Morrison [100].

### NeuN positive cells analysis

Cells were examined for the expression of NeuN. The percentage of NeuN-positive cells was calculated. About 99 neurons, derived from 12 different fields, were analyzed.

### Synaptic marker analysis

The number, size, and colocalization of pre- and postsynaptic puncta were analyzed using Fiji software and the Synapse Counter plug-in for ImageJ, developed by Egor Dzyubenko (Ruhr-University Bochum, Faculty of Biology and Biotechnology, Department of Cell Morphology and Molecular Neurobiology, Head: Prof. Dr. Andreas Faissner) and Andrey Rozenberg. Puncta per 50 µm of neurites and 0.043 mm^2^ area were analyzed.

### Vesicle trafficking analysis (single particle tracking analysis)

Single particle tracking was performed using Fiji software and the Manual Tracking plug-in for ImageJ. Each particle was tracked frame-by-frame to calculate mean velocity; in particular, for reversing behavior, we considered only particles within the initial 20 µm segments of neurites, and the percentage of vesicles, which reverse their trajectory (from anterograde to retrograde) was calculated.

### Mitochondrial morphology analysis

Quantitative image analysis was performed on both Harmony software and the Mitochondrial Analyzer plug-in for ImageJ (Fiji software). Harmony analysis was performed following the procedure previously described by Douida [101]. The Mitochondria Analyzer plug-in was used to confirm results obtained from Harmony, in particular, the parameters taken into account in our study were: mean aspect ratio [(major axis)/(minor axis)], form factor [(perimeter^2^)/(4π·surface area)], branches length/mito (the mean value of the total length of branches of each mitochondrion in a cell), the total area of mitochondria per 0.043 mm^2^ region /n° of cells (the total area of the mitochondria in one cell).

### Neuronal phenotype analysis (ChaT and TH expression and localization)

Quantitative image analysis was performed on both Harmony software and Fiji software. In particular, the ratio between the fluorescence intensity of ChaT and TH in the cell body and ChaT fluorescence intensity in the nuclei were calculated using the Harmony software (cell segmentation was performed based on Hoechst and ChaT or TH staining). Besides, the size and the number of ChaT and TH puncta within neurites (considering an area of 0.043 mm^2^) were analyzed using Fiji software and the Synapse Counter plug-in. To obtain only puncta parameters (number and size) within neurites; before starting the analysis, particles inside cell bodies were manually edited: puncta within the cell body were selected (freehand section) and cleared.

### Statistical analysis

Statistical analysis was performed using GraphPad Prism 8 software. Data were expressed as a mean ± standard error of the mean (SEM). *P* value lower than 0.5 was considered statistically significant (*p < 0.5, **p < 0.01, ***p < 0.001, ****p < 0.0001). *N* value, *p* value, and statistical tests used for each experiment are reported in the corresponding figure legend and in Table 1.

**Table 1:** Data (mean ± S.E.) related to the electrophysiological properties of SH-SY5Y cells in different culture conditions and days of differentiation.

	DIV	I_Na_ (pA/pF)	*N*	I_K_ (pA/pF)	*N*	V_rest_ (mV)	*N*	AP Frequency (Hz)	*N*	Spontaneous Activity (%)	*N*
**2D DMAP1**	5 DIV	153 ± 25	19	108 ± 14	19	−28 ± 2	20	3,6 ± 0,8	18	0%	8
	10 DIV	57 ± 6	13	53 ± 6	13	−24 ± 2	12	1,4 ± 0,4	9	0%	nd
**3D Literature**	10 DIV	115 ± 10	33	120 ± 7	31	−28 ± 1	42	2 ± 0,3	38	0%	10
	20 DIV	114 ± 12	5	97 ± 13	5	−30 ± 4	5	3,2 ± 1,4	5	0%	nd
**3D DMAP1 Coating**	10 DIV	215 ± 31	21	118 ± 11	21	−32 ± 2	21	3 ± 0,4	20	0%	12
	15 DIV	218 ± 36	10	101 ± 15	10	−31 ± 3	9	3 ± 1	9	0%	6
**3D DMAP1 Mix**	10 DIV	187 ± 23	13	118 ± 19	13	−32 ± 4	9	3,5 ± 0,5	10	0%	7
	15 DIV	306 ± 31	20	141 ± 21	20	−39 ± 2	21	4 ± 0,6	21	0%	5
	20 DIV	273 ± 35	12	120 ± 6	12	−45 ± 2	13	5 ± 0,7	14	7%	13
	25 DIV	211 ± 93	4	46 ± 12	4	−39 ± 2	3	4 ± 1	4	0%	3
**3D DMAP2 Mix**	10 DIV	154 ± 32	25	89 ± 12	25	−24 ± 2	29	2 ± 0,5	15	0%	26
	20 DIV	171 ± 45	8	125 ± 24	8	−34 ± 4	12	4,5 ± 0,7	11	12%	8
	25 DIV	275 ± 30	19	134 ± 8	19	−37 ± 3	18	5 ± 0,5	16	25%	16
	30 DIV	392 ± 186	16	102 ± 11	16	−29 ± 2	18	3,7 ± 0,9	14	33%	18
	40 DIV	225 ± 27	12	85 ± 17	12	−30 ± 1	16	4 ± 0,8	15	37%	16

### Supplementary information

Supplemental text and figures

Supplemental video legends

Video 1

Video 2

Video 3

## Data Availability

The authors declare that all data supporting the findings of this study are available within this article, or are available from the corresponding author.
