# Supplementary material for: A new advanced cellular model of functional cholinergic-like neurons developed by reprogramming the human SH-SY5Y neuroblastoma cell line
Source: Cell Death Discov. 2024 Jan 12;10:24. doi: 10.1038/s41420-023-01790-7 (PMC10786877; doi:10.1038/s41420-023-01790-7)
Supplement: Supplementary file 1 — Supplemental text and figures [file 41420_2023_1790_MOESM1_ESM.pdf]

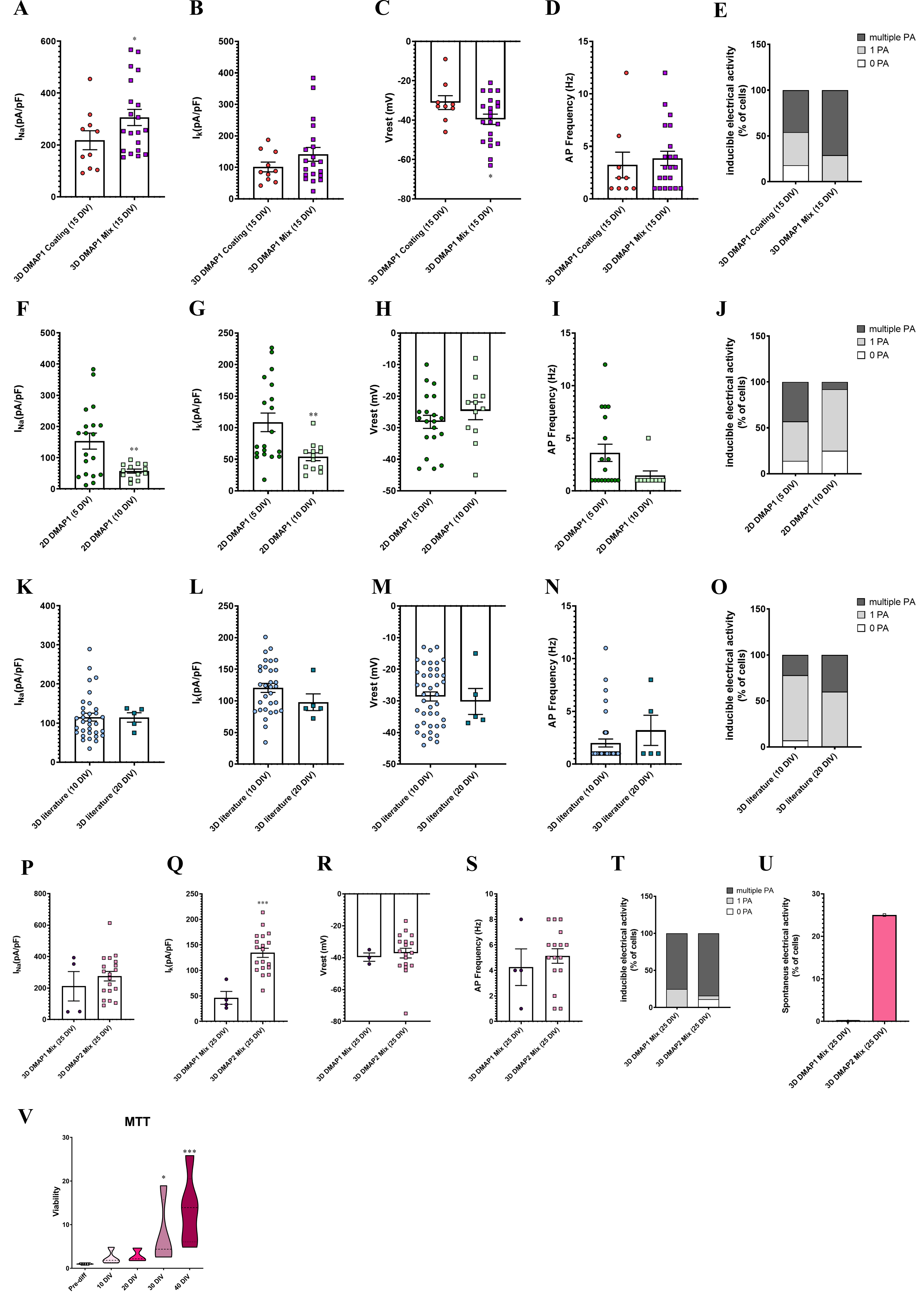

A
B
C
D
E
I_Na(pA/pF)
I_k(pA/pF)
Vrest (mV)
AP Frequency (Hz)
inducible electrical activity (% of cells)
multiple PA
1 PA
0 PA
3D DMAP1 Coating (15 DIV)
3D DMAP1 Mix (15 DIV)
F
G
H
I
J
2D DMAP1 (5 DIV)
2D DMAP1 (10 DIV)
K
L
M
N
O
3D literature (10 DIV)
3D literature (20 DIV)
P
Q
R
S
T
U
3D DMAP1 Mix (25 DIV)
3D DMAP2 Mix (25 DIV)
Spontaneus electrical activity (% of cells)
V
MTT
Viability
Pre-diff
10 DIV
20 DIV
30 DIV
40 DIV

Supplementary Figure 1. Functional properties and viability of SH-SY5Y cells cultured in different conditions at different days of differentiation. We tested sodium and potassium current densities (A, B), resting membrane potential (C), and action potential firing frequency (D, E) of cells cultured in 3D DMAP1 Coating and 3D DMAP1 Mix conditions at 15 DIV. The same parameters were analyzed for the 2D DMAP1 at 5 and 10 DIV (F, G, H, I, J), for the 3D Literature condition at 10 and 20 DIV (K, L, M, N, O), and for the 3D DMAP1 Mix and 3D DMAP2 Mix at 25 DIV (P, Q, R, S, T). The 3D DMAP2 Mix condition showed a higher percentage of cells with spontaneous activity compared to the 3D DMAP1 Mix condition (U). (V) Cell viability (MTT assay) of cells cultured in 3D DMAP2 Mix for 7 days of pre-differentiation, and 10, 20, 30, and 40 days of differentiation (DIV). The number of total samples analyzed for each time point described above were: 5 for Pre-diff., 4 for 10 DIV, 4 for 20 DIV, 4 for 30 DIV, and 5 for 40 DIV. Data are expressed as the ratio between different groups (10, 20, 30, and 40 DIV) and Pre-diff. Differences among groups were tested for significance by: Unpaired t-test, in panels A, C, G, and Q; Chi-Square test, in panel U; Kruskal-Wallis test followed by Dunn's multiple comparisons test, in panel V. Significance was set as $^*p<0.5$, $^{**}p<0.01$, $^{***}p<0.001$, $^{****}p<0.0001$.

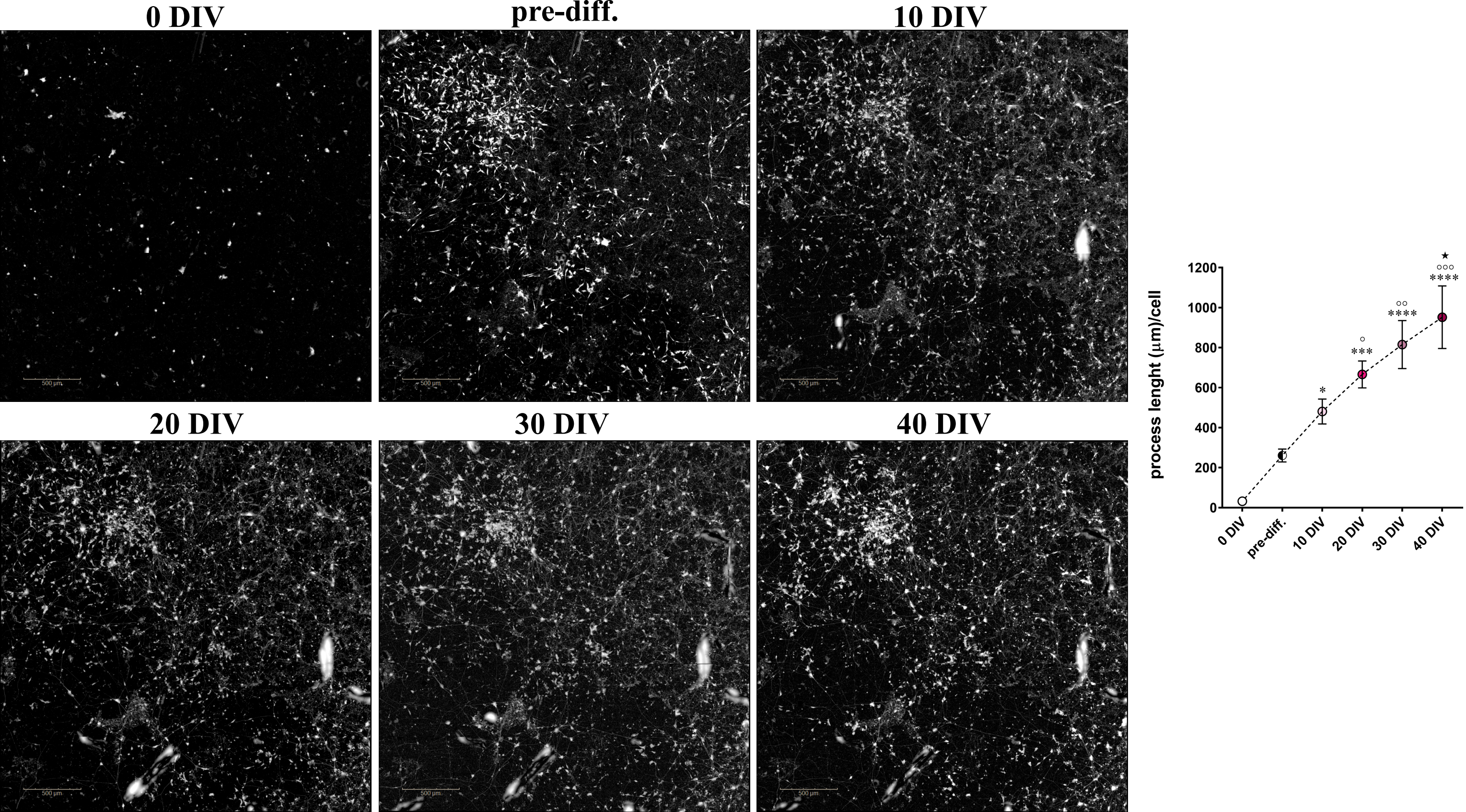
0 DIV
pre-diff.
10 DIV
20 DIV
30 DIV
40 DIV
500 µm
process lenght (µm)/cell
1200
1000
800
600
400
200
0
0 DIV
pre-diff.
10 DIV
20 DIV
30 DIV
40 DIV
*
***
****
****

Supplementary Figure 2. Characterization of neuronal arborization. SH-SY5Y cells were plated and differentiated as described in the Material and Methods section (3D DMAP2 Mix). Live cell images were taken at 0, pre-differentiation (7 days), 10, 20, 30, and 40 DIV using Operetta CLS™ equipped with 20× immersion objective in Digital Phase Contrast (DPC) to detect neurites. Large images (25 fields) are shown in the left panel. Scale bar: 500 µm. Quantification of process length per cell (using Fiji software) is shown in the right panel. 0 DIV, pre-diff., 20 DIV, 30 DIV, and 40 DIV (n=6 large images); 10 DIV (n= 5 large images). One-way ANOVA followed by Tukey's multiple comparisons test was used to determine the significance of differences among the conditions.* indicates significance compared to 0 DIV; ° indicates significance compared to pre-diff; ★ indicates significance compared to 10 DIV. Significance was set as *p<0.5, **p<0.01, ***p< 0.001, ****p<0.0001.

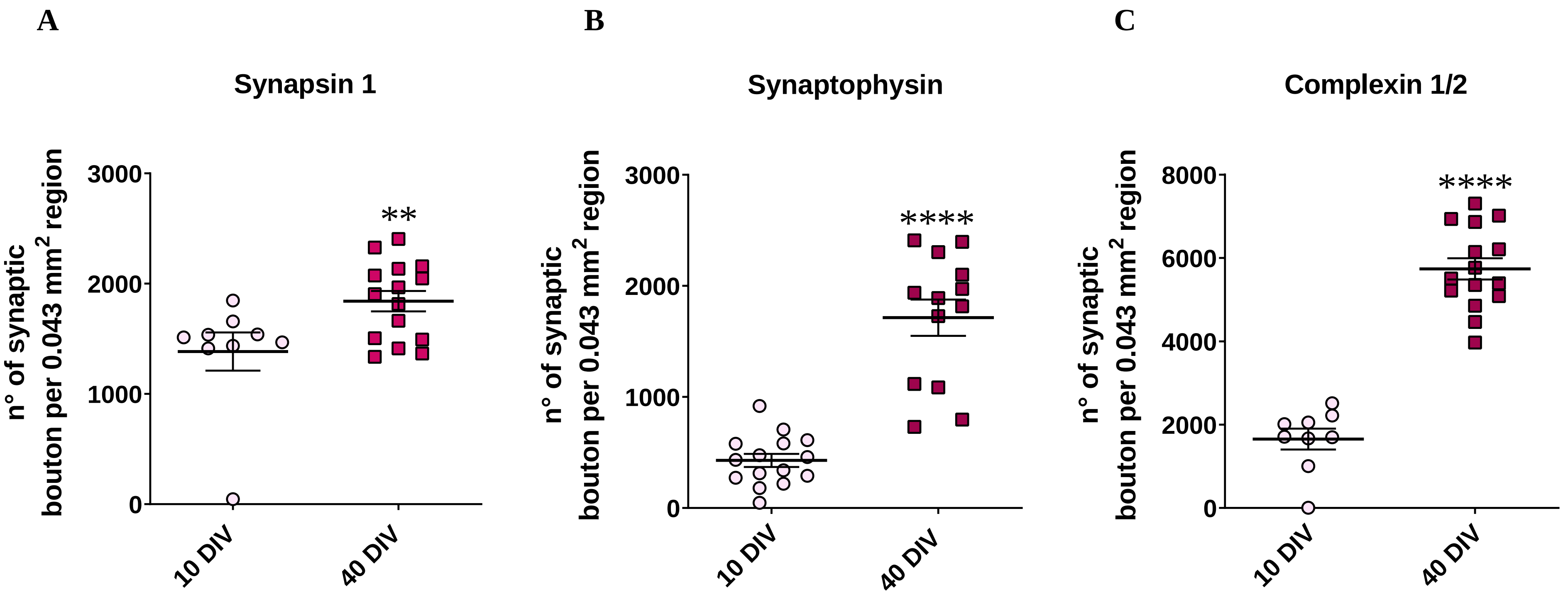
A
Synapsin 1
n° of synaptic bouton per 0.043 mm2 region
3000
2000
1000
0
**
10 DIV
40 DIV
B
Synaptophysin
n° of synaptic bouton per 0.043 mm2 region
3000
2000
1000
0
****
10 DIV
40 DIV
C
Complexin 1/2
n° of synaptic bouton per 0.043 mm2 region
8000
6000
4000
2000
0
****
10 DIV
40 DIV

Supplementary Figure 3. Pre-synaptic markers evaluation. We analyzed the number of pre-synaptic boutons per 0.043 mm^2 region positive for (A) synapsin 1 (10 DIV, n=9 fields, and 40 DIV, n=15 fields), (B) synaptophysin (10 DIV, n=15 fields, and 40 DIV, n=13 fields), and (C) complexin 1/2 (10 DIV, n=9 fields, and 40 DIV, n=15 fields). a), The statistical test used to determine the significance of differences among the conditions was the Unpaired t-test. Significance was set as *p< 0.5, **p<0.01, ***p< 0.001, ****p<0.0001.

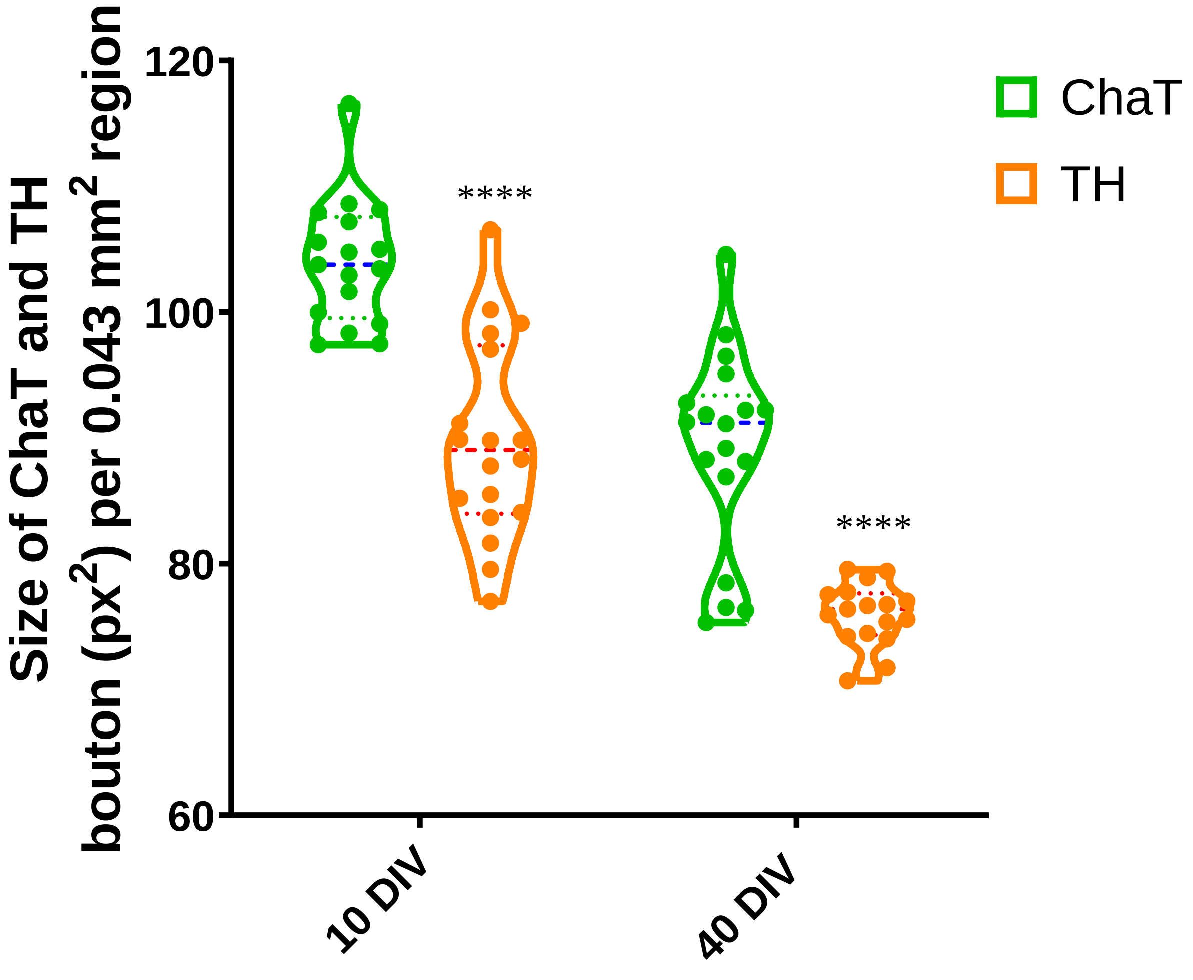

Size of ChaT and TH bouton (px^2) per 0.043 mm^2 region
120
100
80
60
****
****
10 DIV
40 DIV
ChaT
TH

Supplementary Figure 4. Size quantification of ChaT and TH vesicles per 0.043 mm^2 region at 10 DIV (ChaT: n=17 fields, TH: n=18 fields) and 40 DIV (ChaT: n=18 fields, TH:=17 fields). The statistical test used to determine the significance of differences among the conditions was Multiple t-tests. Significance was set as $*p<0.5$, $**p<0.01$, $***p< 0.001$, $****p<0.0001$.
