# Supplementary material for: A new advanced cellular model of functional cholinergic-like neurons developed by reprogramming the human SH-SY5Y neuroblastoma cell line
Source: Cell Death Discov. 2024 Jan 12;10:24. doi: 10.1038/s41420-023-01790-7 (PMC10786877; doi:10.1038/s41420-023-01790-7)
Supplement: Supplementary file 2 — Supplemental video legends [file 41420_2023_1790_MOESM2_ESM.pdf]

Video 1 Time-lapse imaging of living cells at 10 DIV

Video 2 Time-lapse imaging of living cells at 40 DIV

Video 3 Time-lapse imaging of vesicle trafficking at 40 DIV. The red arrow indicates the vesicle which moved with an anterograde trajectory. In contrast, the yellow arrow indicates the vesicle moving with an anterograde trajectory, pausing, and reversing backwards to the soma.
